# Complex Indirect Carotid-Cavernous Fistula With Contralateral Ophthalmic Manifestations

**DOI:** 10.7759/cureus.73670

**Published:** 2024-11-14

**Authors:** Amirah Mohammad Razali, Mohammad Jazli Sobri, Muhammad Khairul Adha Fuad, Anna Misyail Abdul Rashid, Mohamad Syafeeq Faeez Md Noh

**Affiliations:** 1 Ophthalmology, Faculty of Medicine and Health Sciences, Universiti Putra Malaysia, Serdang, MYS; 2 Ophthalmology, Hospital Sultan Abdul Aziz Shah, Universiti Putra Malaysia, Serdang, MYS; 3 Neurology, Faculty of Medicine and Health Sciences, Universiti Putra Malaysia, Serdang, MYS; 4 Radiology, Faculty of Medicine and Health Sciences, Universiti Putra Malaysia, Serdang, MYS

**Keywords:** carotid-cavernous sinus fistula, corkscrew vessels, diplopia, endovascular embolization, proptosis

## Abstract

A carotid-cavernous fistula (CCF) involves an abnormal communication between the carotid artery and the cavernous sinus. For indirect CCF, it usually occurs in post-menopausal women. Contralateral symptoms for indirect CCF are rare. We report a 79-year-old lady with underlying hypertension and dyslipidemia, who had a complex indirect CCF from the right internal and right external carotid artery, draining into the left ophthalmic vein and giving rise to left ocular manifestation. Endovascular embolization was attempted but was unsuccessful. The patient subsequently developed a stroke one day post-procedure, with a favorable recovery of function.

## Introduction

A carotid-cavernous fistula (CCF) is an abnormal communication between the internal or external carotid artery (or its branches) and the cavernous sinus. The cavernous sinus is a venous network containing major neurovascular structures. Among the important structures are the internal carotid artery, the oculomotor nerve, the trochlear nerve, the first and second divisions of the trigeminal nerve, and the abducent nerve. There are a few classifications for CCF. It can be classified based on the cause, either spontaneous or traumatic, based on hemodynamic properties, which may be low flow or high flow, or by the angiographic classification, which can be direct or indirect [1]. Traumatic CCFs are more common, comprising 75% of cases, while the remaining are spontaneous CCFs occurring commonly in older ladies [2].

The eye is a common initial presentation for patients with CCF. Dandy’s triad includes proptosis, chemosis, and a bruit, occurring usually in direct CCFs [1]. Other ocular signs are pulsatile proptosis, corkscrew vessels, increased intraocular pressure, optic disc swelling, and central retinal vein occlusion [3]. Usually, these signs and symptoms occur on the ipsilateral side of the CCF as, usually, the carotid artery will drain to the ipsilateral cavernous sinus and is only connected to the contralateral side via the anterior and posterior intercavernous sinuses across the midline. Rare presentations of contralateral symptoms have been reported in post-traumatic cases and cases with primitive persistent trigeminal arteries [4,5]. We report a case of a complex spontaneous indirect CCF with contralateral ocular manifestations.

## Case presentation

**Figure 1:**
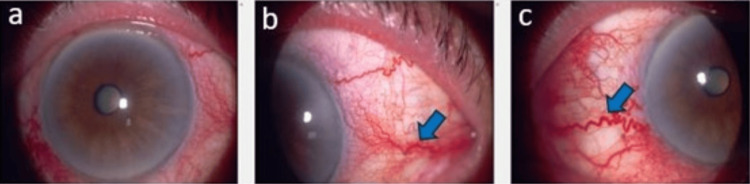
Anterior segment photo of the left eye showing the presence of corkscrew vessels (blue arrow) over the temporal (b) and nasal (c) conjunctiva.

A 79-year-old woman with underlying hypertension and dyslipidemia presented with a one-month history of left eye redness associated with diplopia, blurring of vision, and headache. There was no prior history of trauma. She was already on two fixed combination eye drops, which were travoprost 0.004% and timolol 0.5% daily and also brinzolamide 1% and brimonidine tartrate 0.2% twice daily over the left eye, which was started at another center. On examination, the best corrected visual acuity was 6/7.5 for the right eye and 6/9 over the left eye. The relative afferent pupillary defect was negative. There was left eye proptosis with limited elevation. The anterior segment examination of the right eye was unremarkable with early nuclear sclerosis and intraocular pressure of 14 mmHg. For the left eye, the conjunctiva was injected with corkscrew vessels present nasally and temporally (Figure 1).

**Figure 2:**
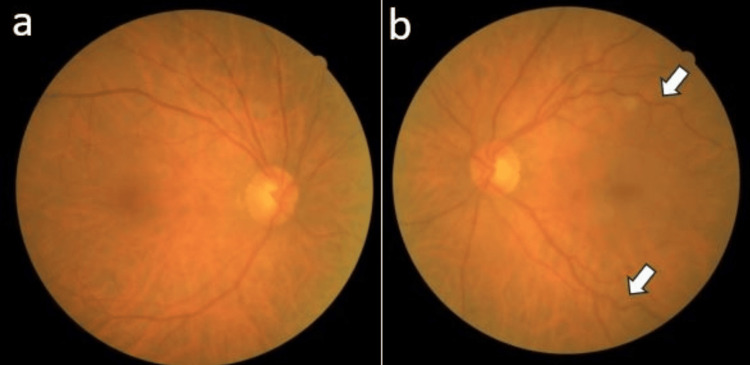
Fundus photo of the right eye (a) and the left eye (b) showing the presence of tortuous veins over the left eye (white arrow).

The cornea was clear with no anterior chamber activity. There was early nuclear sclerosis with an elevated intraocular pressure of 24 mmHg while on the two fixed combination antiglaucoma eye drops. The right fundus examination was unremarkable with a cup-to-disc ratio of 0.4 and a normal macula. The left eye fundus examination revealed a pink optic disc with a cup-to-disc ratio of 0.4. The veins were slightly tortuous but not dilated, and there was an epiretinal membrane over the macula (Figure 2).

**Figure 3:**
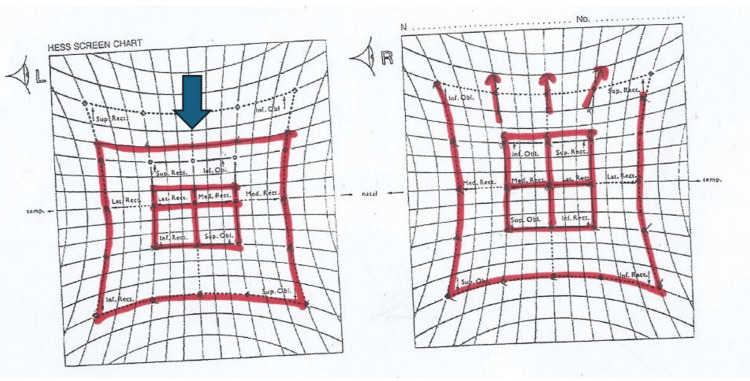
HESS chart examination at presentation showing the under-action of the left superior rectus muscle (blue arrow).

The HESS chart (Figure 3) revealed the under-action of the left superior rectus muscle while the Humphrey visual field was normal bilaterally.

**Figure 4:**
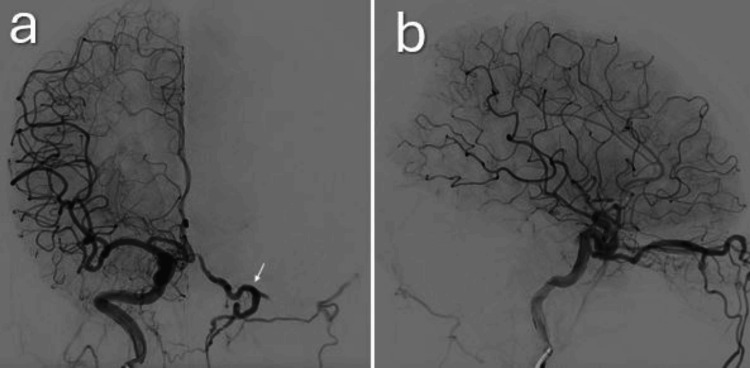
Right internal carotid artery angiographic run both frontal (a) and lateral (b) views showing a complex indirect carotid-cavernous fistula (CCF), with drainage into the left venous system. The complex indirect CCF with engorged left ophthalmic vein is seen (white arrow).

A cerebral angiogram revealed a complex indirect CCF from the right internal and right external carotid artery, draining into the left venous system (Figure 4).

Subsequently, the patient underwent endovascular embolization, but during the procedure, no obvious communication was found between the left superior ophthalmic vein and the cavernous sinus. The arterial feeder of the left superior ophthalmic vein was from the middle meningeal artery, which was unable to be cannulated due to its small size and tortuosity. Unfortunately, one day after the procedure, the patient developed right upper limb weakness with right facial asymmetry. Magnetic resonance imaging of the brain revealed an acute multifocal infarct involving the bilateral frontal-temporal-parietal region with no evidence of intracranial bleed. She was not keen on other interventions thereafter. Her eyes remain stable with the current topical antiglaucoma treatment. Her latest visual acuity maintained at 6/7.5 for the right eye and 6/9 over the left eye. The left intraocular pressure ranged between 22 and 24 mmHg, and there were no glaucomatous visual field changes seen. She is being closely monitored and co-managed with the glaucoma team for which surgical intervention has been planned if the intraocular pressure further rises or if there is evidence of any glaucomatous changes seen. Her ocular motility remained the same with limited elevation.

## Discussion

A CCF is a rare condition that may cause devastating ocular complications. Barrow et al. categorized CCFs into four types based on the arterial supply [6]. Type A is high flow whereby there is direct communication between the ICA and the cavernous sinus [6]. Types B, C, and D are low-flow fistulas. A type B fistula has dural ICA branches communicating with the cavernous sinus, while type C fistula has dural branches of the ECA communicating with the cavernous sinus and type D fistula in which the dural ICA and ECA branches communicate with the cavernous sinus [6]. In our case, the patient had right-sided type D indirect CCF. When CCF occurs, highly pressurized arterial blood enters directly into the venous system causing an increase in venous pressure. The clinical manifestations of CCFs are due to the elevation in the intracavernous pressure and revised flow patterns [1]. The symptoms include eye redness, diplopia, orbital or retroorbital pain, swishing sound, headache, and blurring of vision [1,3]. Ocular signs are proptosis, cephalic bruit, oculomotor nerve and abducent nerve palsy, conjunctival chemosis, corkscrew vessels, raised intraocular pressure, exposure keratopathy, neovascular glaucoma, angle closure glaucoma, venous stasis, retinal hemorrhages, central retinal vein occlusion, and optic disc swelling [1,3].

Ocular manifestation usually occurs on the ipsilateral side. There are only a few cases of CCF with contralateral ocular manifestation. For post-traumatic cases causing direct CCF, Zhu et al. reported a defect of the intracavernous segment of the right ICA with subsequent flow of high-pressure arterial blood to the contralateral cavernous sinus leading to left ocular symptoms [4]. Another post-traumatic cause was reported by Hoang et al. in which a left CCF induced contralateral ocular symptoms via the intercavenous sinus segment but related to persistent primitive trigeminal artery [5]. For the spontaneous CCF, Blasi et al. reported an elderly gentleman with left ocular symptoms who was found to have CCF in which the feeder was the meningo-hypophyseal branches originating from the right ICA [7]. Although the other reported cases were successfully treated with endovascular embolization, due to the complexity of our case, the procedure was unsuccessful in our patient due to challenging access to the fistula. While an alternative approach was planned and suggested for the patient, she was not willing to proceed with further interventions due to the complication of stroke that she had after the first procedure.

The aim of treatment for CCF is fistula occlusion whilst ensuring a usual flow through the ICA [2]. Endovascular treatment, either via transarterial or transvenous approach is usually the first line of treatment in the majority of cases. Detachable balloons or metallic coils and/or liquid embolic agents are usually used to occlude the fistula [1,2]. A conservative approach may be chosen for patients with mild symptoms or low-risk lesions. External manual compression of the ipsilateral carotid artery a few times a day may be effective for indirect, low-flow fistulas [2]. Spontaneous resolution may occur within days to months after the initial presentation due to further thrombosis of the involved segment of the cavernous segment [1]. Indications for emergency treatments can be divided based on angiographic features or based on clinical signs and symptoms. For angiographic findings, indications include pseudoaneurysm, large varix of the cavernous sinus, venous drainage of cortical veins, and thrombosis of distant venous outflow pathways [1]. For clinical manifestations, indications include rapidly progressive proptosis, raised intracranial pressure, intracranial bleeding, and transient ischemic attack [1]. Around 20-30% of dural CCF leads to poor vision, mainly due to uncontrolled glaucoma or ischemic neuropathy [3]. Common complications of CCF include exposure keratopathy due to proptosis, ocular hypertension or glaucoma, and ischemia leading to proliferative retinopathy. For patients with exposure keratopathy, regular lubricants are essential to maintain the integrity of the corneal surface. Tarsorrhaphy may also be an option to help protect the cornea. Elevated intraocular pressure in CCF may be due to a few mechanisms. Common causes include raised episcleral venous pressure and orbital congestion. It may also be due to angle closure and choroidal detachments. The other mechanism is due to ischemia, leading to rubeosis and neovascular glaucoma. Topical antiglaucoma is usually adequate to control the intraocular pressure, especially those that act to reduce aqueous production, but in some refractory cases, systemic antiglaucoma medication and glaucoma filtration surgery may be needed to save the eye from glaucomatous damage [8]. A peripheral iridotomy may be needed for those with angle closure and those with evidence of ischemia will need pan-retinal photocoagulation.

Optical coherence tomography is a convenient and non-invasive tool for the follow-up of CCF patients. Yonca et al. found that enhanced depth imaging spectral domain optical coherence tomography (SD-EDI-OCT) can be used to measure choroidal thickness, choroidal vascularity index, and tortuosity index to evaluate the occlusion of fistulas either spontaneously or by endovascular treatment [9]. Interestingly, they have demonstrated that the contralateral clinically unaffected eye also shows an increase in subfoveal retinal and choroidal thickness and a change in choroidal vascularity index [9]. Overall, there is a reduction in the choroidal thickness, choroidal vascularity index, and tortuosity index after treatment or spontaneous closure of the fistula [9]. It is also worth noting that in some cases, patients may experience transient worsening of the ocular condition after the closure of the fistula, commonly occurring immediately after closure but may rarely occur after a few months [10]. It has been postulated that thrombosis of the superior ophthalmic vein with insufficient collateral drainage leads to worsening of the signs and symptoms after which once collaterals develop or expand, there will be improvement and resolution of the ocular manifestation [11]. Hence, it is vital that ophthalmologists closely monitor patients with CCF before and after treatment as the ocular condition may deteriorate rapidly. Failure to commence appropriate treatment will lead to devastating ocular complications and may lead to blindness.

## Conclusions

A complex indirect CCF may lead to contralateral ocular manifestation in which the embolization process may be challenging and cause significant morbidity to the patient. Since these patients frequently present to the ophthalmologist, accurate diagnosis is essential to allow further appropriate management of the patient. A multidisciplinary approach is core in managing these complex cases to ensure favorable outcomes.
